# Highly-integrable analogue reservoir circuits based on a simple cycle architecture

**DOI:** 10.1038/s41598-024-61880-z

**Published:** 2024-05-14

**Authors:** Yuki Abe, Kazuki Nakada, Naruki Hagiwara, Eiji Suzuki, Keita Suda, Shin-ichiro Mochizuki, Yukio Terasaki, Tomoyuki Sasaki, Tetsuya Asai

**Affiliations:** 1 Graduate School of Information Science and Technology, Hokkaido University, Kita 14, Nishi 9, Kita-ku, Sapporo, Hokkaido 0600814 Japan; 2 Advanced Products Development Center, Technology and Intellectual Property HQ, TDK Corporation, 2-15-17 Higashi-Owada, Ichikawa, Chiba 2728558 Japan; 3 Faculty of Information Science and Technology, Hokkaido University, Kita 14, Nishi 9, Kita-ku, Sapporo, Hokkaido 0600814 Japan

**Keywords:** Physical reservoir computing, Analogue circuit, Edge computing, Chaos prediction, Computer science, Electrical and electronic engineering

## Abstract

Physical reservoir computing is a promising solution for accelerating artificial intelligence (AI) computations. Various physical systems that exhibit nonlinear and fading-memory properties have been proposed as physical reservoirs. Highly-integrable physical reservoirs, particularly for edge AI computing, has a strong demand. However, realizing a practical physical reservoir with high performance and integrability remains challenging. Herein, we present an analogue circuit reservoir with a simple cycle architecture suitable for complementary metal-oxide-semiconductor (CMOS) chip integration. In several benchmarks and demonstrations using synthetic and real-world data, our developed hardware prototype and its simulator exhibit a high prediction performance and sufficient memory capacity for practical applications, showing promise for future applications in highly integrated AI accelerators.

With the development of the information society, collecting various time-dependent data such as consumption trends, stock prices, climate variations, and confirmed disease cases, has become increasingly straightforward. Artificial intelligence (AI), significantly advanced through artificial neural networks (ANNs), facilitates the prediction of future trends and the discovery of unknown rules underlying these data. Although the AI demand is projected to increase, current software-based AI faces challenges in power consumption and processing speed due to the von Neumann bottleneck. In particular, cloud-based AI computing, the prevalent technology in AI, demands significant electricity, computing resources, and data traffic^1,2^.

To address these challenges, various types of hardware dedicated to ANN calculations have been proposed to accelerate AI processing. In particular, physical reservoir computing (PRC), which exploits various physical systems and materials with nonlinear dynamics, has attracted significant attention in recent years. RC is a framework for computation derived from recurrent neural network in which input data are transformed nonlinearly via a recurrent network called a reservoir, and are capable of processing time-dependent data, such as dynamical prediction and time-dependent data classification^3–7^. The output of reservoir is obtained through a readout unit in which the weighted linear sum of the reservoir node states is obtained (see Eq. (2) in Methods section for more details). Training of the reservoir can only be implemented by training the readout, resulting in reduced computing and memory resources. In the PRC framework, reservoirs can be replaced by physical systems with nonlinearity, fading memory, high dimensionality, and consistency. For example, optical devices^8–11^, electric circuits^12–15^, spintronic devices^16–18^, nano materials^19,20^, quantum systems^21^, and soft robots^22^ have been proposed as promising physical reservoirs for hardware acceleration in AI computing. The top–down approach of adopting promising physical systems such as reservoirs and configuring the entire RC system has recently become mainstream in a wide variety of research fields.

Designing and controlling the selected physical system as a reservoir presents significant challenges in the top–down approach. In addition, the low reliability and integration remain challenging, making it difficult to apply edge AI computing. Based on this background, we propose a physical reservoir using a bottom–up approach, in which analogue circuits were designed based on an architecture called a simple cycle reservoir (SCR), and the performance of the developed hardware prototype was evaluated through benchmark tasks. The proposed reservoir was designed using metal-oxide-semiconductor field-effect transistors (MOSFETs), resistors, capacitors, and sparse wires. These components can be integrated onto standard complementary metal-oxide-semiconductor (CMOS) chips as silicon integrated circuits. Consequently, the proposed architecture is well-suited for CMOS chip integration, as silicon CMOS integrated circuits (chips) play a crucial role in various edge computing devices.

Notably, the proposed physical reservoir has a remarkable memory capacity (MC), indicating its high capability for processing time-dependent data. To support this, the prediction of time-dependent data, such as synthetic chaotic oscillations and the transition of confirmed cases of the infectious disease COVID-19, which has had a significant impact on society in recent years, was demonstrated.

Moreover, the proposed physical reservoir has high compatibility with conventional digital circuits because the circuit can be implemented on CMOS chip, and the proposed architecture has a serialized input and output (I/O) interface. Since edge computing devices have low-level (and thus low power) serial I/O interfaces, the proposed architecture can be connected to them without any hardware overhead. Therefore, when the architecture is integrated onto a chip, the incorporation of edge devices and the reservoir chip becomes highly feasible. Furthermore, online learning architecture for physical reservoirs having digital serial I/O interfaces has already been established for the integration with reservoir hardware^23^. Time series prediction based on online learning at the edge has been impractical due to the power and resource demands of existing digital systems. However, our architecture enables its implementation, ushering in a new field of edge AI applications.

## Results

### Concept of analogue SCR circuit

An echo state network (ESN) serves as the primary model for RC. In an ESN, a substantial number of nonlinear nodes within a reservoir are interconnected randomly with fixed weights The complex dynamics of the reservoir enable the ESN to nonlinearly map input data to higher-dimensional spaces, analogous to the function of the cerebellum in the brain, which is composed of non-plastic synapses^24^. However, emulating an ESN with a complex random network using analogue circuits results in increased complexity in the circuit configuration and an enlarged circuit area. Based on a performance comparison between several device-friendly reservoir models, including delay-based reservoir systems (with and without feedback) and so on, we selected a simple-cycle reservoir (SCR)^25^ as our implementation target because the sparsity of SCR’s connection is conducive to electrical circuits, as compared to ESN, while still maintaining better performance for the given dataset when compared to delay systems. This choice aligns with our goal of achieving efficient reservoir computing using CMOS-friendly devices.

**Figure 1:**
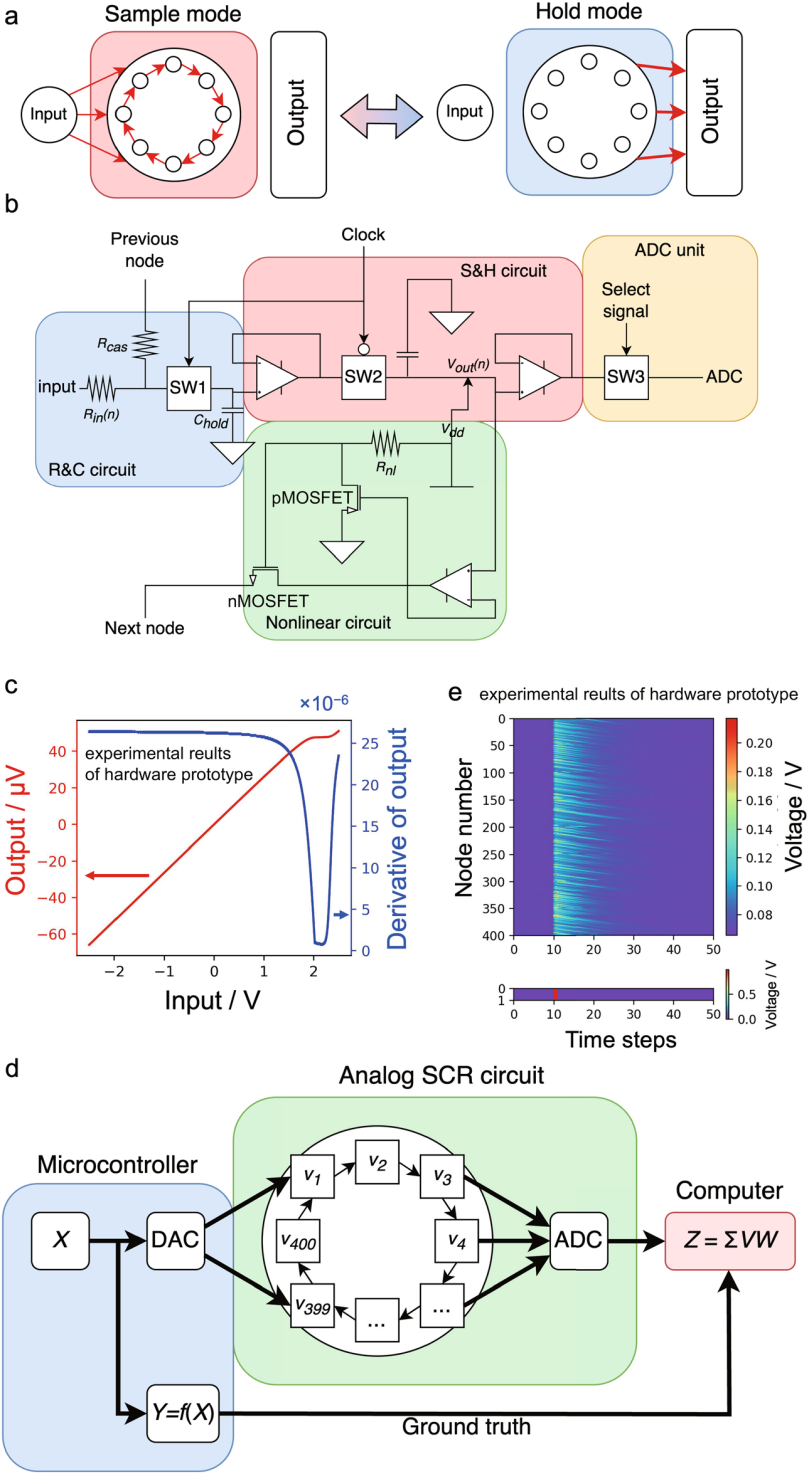
Concept and basic characteristics of the developed PRC system. (**a**) Schematics depicting the switch between sample and hold modes in the physical reservoir computing (PRC) system. In sampling mode, the input signal is cyclically circulated in the reservoir, updating the state of each node. In hold mode, each node’s state is read to derive the RC output. (**b**) Circuit diagram of a nonlinear node circuit composed of a resistor–capacitor (R and C) circuit, a sample and hold (S and H) circuit, nonlinear circuit, and analogue-to-digital converter ADC unit. (**c**) Input–output characteristics of the nonlinear circuit, displaying the output voltage and its derivative’s dependence on input voltage as red and blue lines, respectively. (**d**) Schematic of the entire PRC system. Input *X* generated by the microcontroller is fed to the analogue simple cycle reservoir (SCR) circuit, and the potential $V_n$ of each nonlinear node circuit is updated and read out as a reservoir state. RC output *Z* is calculated on software using the reservoir states *V* and readout *W*. (**e**) Impulse response of the prototype hardware, demonstrating signal transmission between neighboring nodes and circulation in the analog SCR circuit.

In the SCR architecture, the input is provided to all nonlinear nodes, which are circularly connected to each other within the reservoir. The nodes dynamically change their state by transmitting their own state to neighboring nodes unidirectionally. In this study, the *n*-th node (where $n = 1, 2, \cdots , N$) is represented by a node circuit, with its state at a discrete time step $\tau$ being expressed as the electrical potential $V_{n, \tau }$. Here, *N* represents the total number of nodes. Figure 1a shows a schematic of the operations of the proposed analogue SCR circuit, wherein the sampling and holding operations are switched by sample and hold (S and H) circuits at each time step. In sampling mode, the data in the SCR circuit are circulated, and each node state $V_{n, \tau }$ is updated. In the holding mode at $\tau$, each node state $V_{n, \tau }$ is held and read.

### Nonlinear node circuits

One of the important circuit in each node of our physical reservoir is a resistor–capacitor (R and C) circuit, characterized by resistance $R_{in}$ and capacitance *C*. The circuit is introduced for physical implementation of echo state property (ESP) of reservoirs^3^. The R and C circuit shows time-dependent input–output characteristics, which are described as follows:

1 $$\begin{aligned} V_{out}(t) = V_{in}+(V_{out}(0)-V_{in})e^{-\frac{t}{CR_{in}}} \end{aligned}$$

Here, *t* represents the real-time, $V_{in}$ is the input voltage to the R and C circuit, and $V_{out}(t)$ denotes the potential of the capacitor. From Eq. (1), it is apparent that $V_{out}(t)$ fades over time when $V_{in}=0$ because capacitor *C* is discharged by resistor $R_{in}$. Hence, the R and C circuit works as a fading memory. This fading property ensures that only short-term memory information is utilized, making reservoirs computationally efficient^3^. Feasible study of PRC using the R and C circuits as reservoir nodes is discussed in the Supplementary Information.

The linear response represented by Eq. (1) is insufficient as a reservoir node because most real-world data in society are generated by complex nonlinear dynamics. Hence, a nonlinear circuit based on a horizontal resistor circuit^26^ was developed and combined with an R and C circuit to provide nonlinearity to the node circuit. The design of the nonlinear node circuit is illustrated in Fig. 1b, which is composed of the R and C circuit, nonlinear circuit, S and H circuit, and analog-to-digital converter (ADC) unit. The $n\text {th}$ node circuit receives the input signal and the signal from the $(n-1)\text {th}$ circuit via resistors $R_{input}$ and $R_{cas}$ at the R and C circuit unit, respectively. The proposed nonlinear circuit, characterized by a straightforward configuration, functions as a means of nonlinearly transmitting the state of the $n\text {th}$ node to the subsequent $(n+1)\text {th}$ node. Figure 1c shows the input–output characteristics of the nonlinear circuit, indicating a nonlinear property that corresponds to a half-sigmoidal function that returns nonlinear output only for positive input. The configuration of the nonlinear circuit is detailed in the Supplementary Information.

The S and H circuit was introduced to switch between the sample and hold modes, and it was controlled by a clock signal and two analogue switches $SW_1$ and $SW_2$: the high state of the clock signal switches to the sample mode and the low state switches to the hold mode. The frequency of the clock signal generated by the microcontroller was fixed at 250 Hz, enabling the system to alternate between the two modes every 2 ms. During the sample mode, a hold capacitor with a capacitance of $C_{hold}$ charges, and its potential is dynamically updated based on the past input. During the hold mode at a discrete time step $\tau$, the potential of the capacitor in the $n\text {th}$ node circuit is held by the S and H circuit, and the held potential $V_{n, \tau }$ is read out as a node state by the ADC unit. In this study, $N=400$ was adopted as the total number of nodes.

### Configuration of hardware prototype PRC system

Based on the above, a hardware prototype of the PRC system was developed. The prototype is illustrated in Fig. S1. The prototype is composed of eight printed circuit boards (PCBs) and a microcontroller. The settings of various parameters such as resistance and capacitance are detailed in the Methods section. Since using an ADC for each node circuit requires more logic circuits and makes the entire system more complex, the ADC circuit was sheared with 50 node circuits on a board. In the hold mode, the states of 50 node circuits were read out using a time-division multiplexing approach, and an analog switch $SW_3$ was controlled to select the nonlinear node circuit for access.

A schematic of the proposed PRC system is presented in Fig. 1d. First, the microcontroller generates an input sequence $X = {x_0, x_1,..., x_\tau ,...}$ and converts it into an analogue voltage sequence in the range of −1 to 1 V using a digital-to-analog converter (DAC). The input is fed to the analogue SCR circuit and circulated. The obtained node states were recorded and processed using a software to calculate the RC output. The inference and training of RC in the software are detailed in the Methods section.

For an operational test, a 1 V voltage impulse was input to the hardware prototype, and the output from each node circuit was sampled. The responses obtained for the input impulses are presented in Fig.1e, in which the impulse was applied at $\tau = 10$. It can be observed that the input impulse to a node propagates to neighboring nodes one after another with attenuation, showing the capability of retaining input data as fading memory.

**Figure 2:**
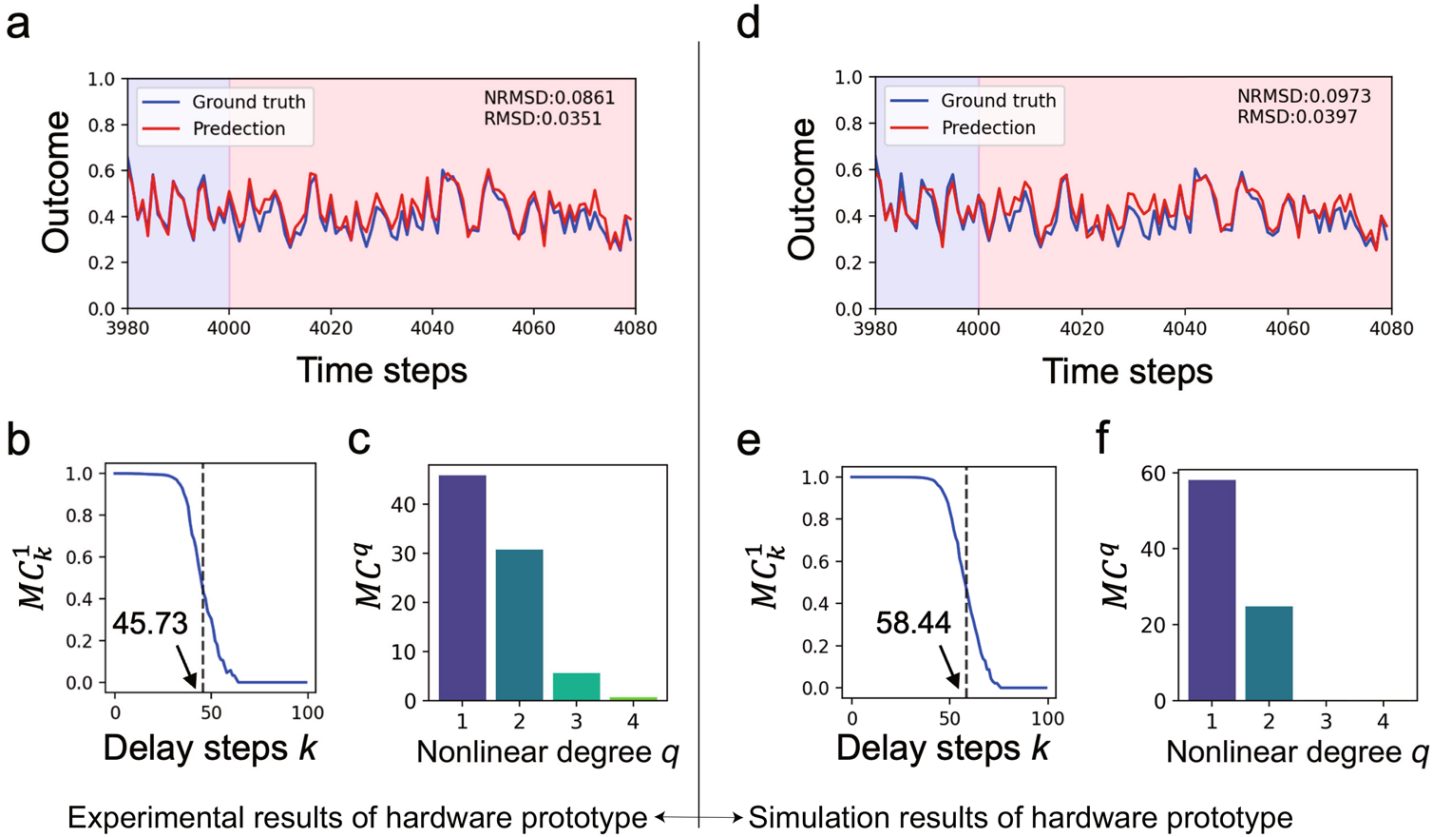
RC performance comparison between the (**a**–**c**) hardware prototype and (**d**–**f**) simulator through typical benchmark tasks. (**a**, **d**), (**b**, **e**), and (**c**, **f**) indicate the results of NARMA10 prediction, linear MC at each time step *k*, and nonlinear MC of nonlinear degree *q*, respectively.

### Performance evaluation through benchmarks

The performance of the prototype was evaluated using several benchmark tasks. First, we attempted to predict the tenth-order nonlinear autoregressive moving average (NARMA10), which is often used as a benchmark to measure RC performance^27^. NARMA10 is a supervisor signal generated from an input sequence and behaves chaotically depending on the input up to 10 time steps ago; therefore, it is often used to evaluate the long-term fading-memory property of the reservoir. In this benchmark, a uniform random input sequence with 5000 discrete time steps was generated in the range of −1 to 1 and normalized to the range 0.0 of 0.5. NARMA10, the ground truth, was obtained from the generated input based on the equation described in the Methods section. The experiment contained three phases: initialization of 1000 time steps, training of 3000 time steps, and evaluation of 1000 time steps. During the initialization, the input is fed to the analogue SCR circuit to stabilize its dynamic behavior. Subsequently, the RC output sequence and the error with the ground truth were calculated, and the readout was trained using the software. Finally, the RC output sequence was obtained using the trained readout, and the prediction errors were calculated during the evaluation. The root mean square error ( RMSE) and normalized RMSE ( NRMSE) served as measures of prediction error (for detailed information, refer to the Methods section). The results of NARMA10 prediction task using the hardware prototype PRC system are shown in Fig. 2a. The red line indicates the prediction from the prototype and the blue line indicates the ground truth. The pale blue and red areas indicate the training and evaluation phases, respectively. The prediction was generated accurately even during evaluation, with NRMSE = 0.0861 and NMSD = 0.0351, showing excellent prediction performance as a physical reservoir. These errors are comparable to those of several previously proposed physical reservoirs (see Supplementary Information).

To evaluate the fading-memory characteristics of the developed prototype, linear, and nonlinear MC, which quantitatively represent how much past input data transformed linearly and nonlinearly can be retained in the reservoir^28^ as a memory, were obtained (see Methods section for details on calculations). In this benchmark, the linear/nonlinear MC ($MC_k^q$) for the input before *k* steps was measured, where *q* denotes the degree of nonlinearity. Figure 2b shows the curve obtained with linear MC ($MC_k^1$) for each delay step $k (= 0, 1, 2, ..., 100)$. The overall linear MC $(\equiv MC^1 = \sum _{k=0}^{100} MC_k^1$) defined as the area under the curve in Fig. 2b, was calculated as 45.73 and shown by the dotted line in the figure. The obtained $MC^1$ is an excellent physical reservoir value (see Supplementary Information), indicating that the prototype system is applicable to time-dependent data processing that requires long-term fading memory.

Nonlinear MC $(MC^q, q = 2, 3, 4,...)$ can also be obtained using a procedure similar to that for linear MC ($MC^1$, see Methods for more details). Figure 2c shows the obtained linear/nonlinear MCs of degrees 1, 2, 3, and 4, with values of 45.12, 30.62, 5.46, and 0.57, respectively, indicating that linear and quadratic MCs are dominant.

To accelerate evaluation of the proposed PRC system, a simulator of its equivalent circuit was constructed using NGSPICE^29^. Figure 2d and e show the results of the NARMA10 prediction and the linear MC obtained from the simulator, in which NRMSE and linear MC were calculated as 0.0973 and 58.44, respectively. Figure 2f shows the obtained linear and nonlinear MCs, in which those of degrees 1 and 2 are 57.92 and 24.65, respectively, and above degree 3 were nearly zero. The simulation results mostly reproduced the performance of the hardware prototype with abundant linear and quadratic MCs. The benchmark results are summarized in Table 1.

**Table 1:** Summary of benchmark results.

	MC of degree				NARMA10 prediction	
	1	2	3	4	RMSE	NRMSE
Hardware prototype	45.12	30.62	5.46	0.57	0.0351	0.0861
Simulation	57.92	24.65	0.00	0.00	0.0397	0.0973

**Figure 3:**
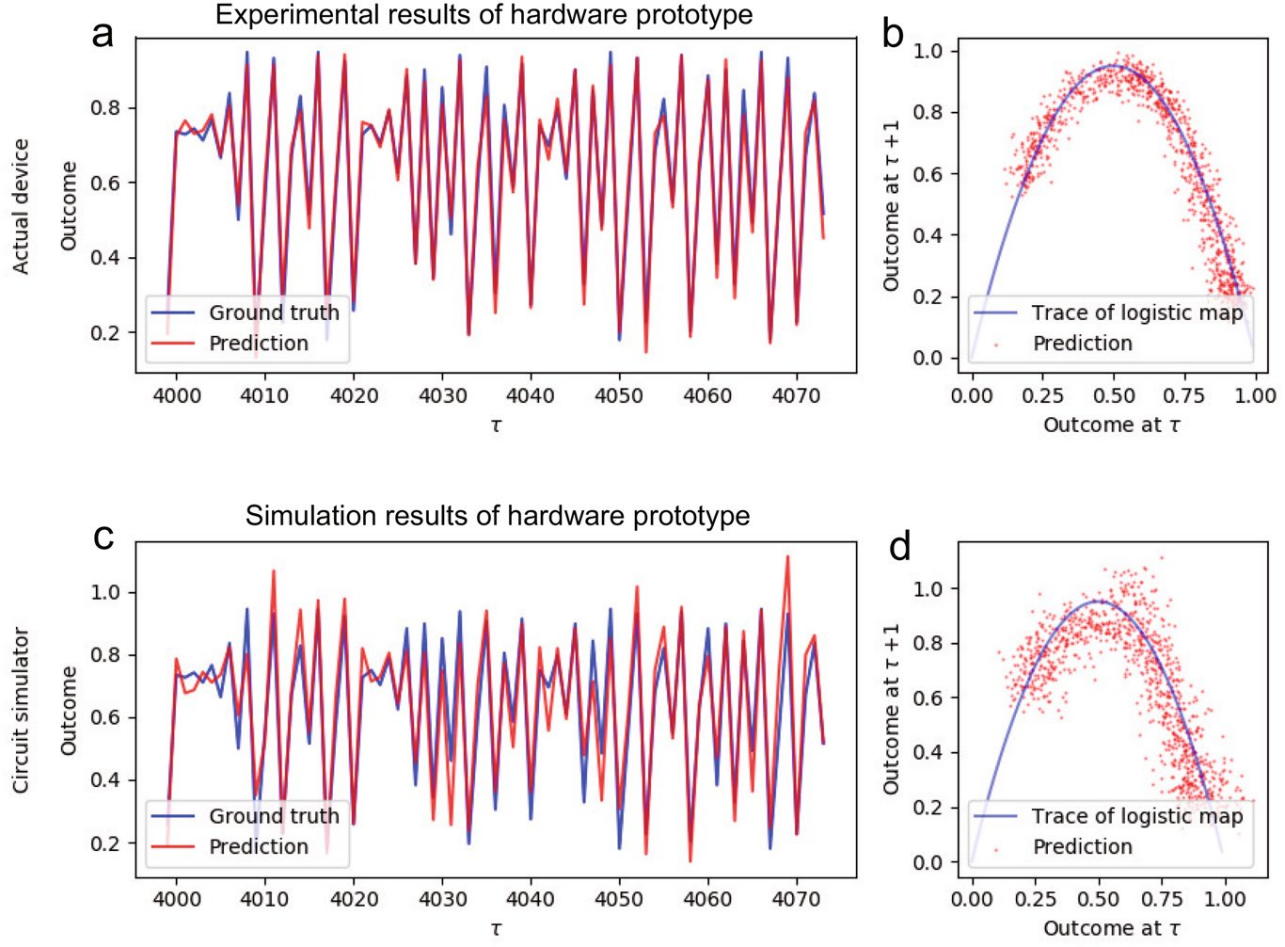
Logistic map prediction results. Experimental results of (**a**) time transition and (**b**) return map of the logistic map predictions by the developed PRC hardware prototype. Simulation results of the hardware are shown in (**c**) and (**d**). Here, the blue line represents the ground truth, while the red line or dots represent the prediction results.

### Demonstration of synthetic data prediction

The prediction of a chaotic system was attempted using the developed prototype. Here, a logistic map, which is known as a chaotic system that can arise from simple nonlinear dynamic equations, was adopted as the prediction target, and the RC input $x_{\tau +1}$ at time step $\tau$+1 was generated based on the equation $x_{\tau +1} = ax_\tau (1-x_\tau )$, where *a* is a parameter that regulates the dynamics of the system. In this study, *a* and the initial input value $x_0$ were set to 3.8 and 0.4, respectively, and the input transition exhibited chaotic behavior^30^. The objective of this task was to precisely predict the input at the next time step, and the prediction of $x_{\tau +1}$ from $x_{\tau }$ was attempted at time step $\tau$. Figure 3a shows the experimental results of the hardwarel prototype, where the red and blue lines indicate the prediction results and their ground truth during the evaluation. NRMSE between the prediction and ground truth was 0.0518, indicating that the prototype could accurately predict the chaotic behavior accurately owing to its rich MC of quadratic nonlinearity, which is required for predicting the logistic map. The return map displayed in Fig. 3b, illustrating the connection between a predicted value at one time step and the next, suggests that the quadratic nonlinear equation underlying the logistic map could be precisely approximated.

Simulation results of the hardware are shown in Fig. 3c and d for the comparison. The results showed that the hardware exhibited better performance as compared to simulations (NRMSE: 0.1089). This is because the discrete MOSFET’s device variation in the nonlinear circuit (Fig. 1b) cannot be modeled in the simulations precisely due to the lack of the variation data in the device specifications (see Method section for used MOSFETs), while our intentional variations of the resistors in the R and C circuit were modeled precisely, which suggest that the MOSFET’s variation plays important role for the reservoir’s performance. Therefore, for the CMOS chip implementation, MOSFETs must be designed to reduce unpredictable device variations by using large channels, and the channel dimensions (channel width and length) should be intentionally designed to have desired variations, through optimization with extensive numerical simulations.

These findings, based on synthetic data, demonstrate the potential of the developed PRC system for predicting chaotic systems in real-world applications.

### Demonstration of real-world data prediction

In a more complex demonstration, real-world data prediction was attempted: a daily forecast of confirmed COVID-19 cases, a task of increasing societal importance^31^. More recently, many studies have been conducted on controlling situations from a statistical perspective using machine learning^32–34^. For instance, it has been reported that RC is effective for daily ahead forecasting, in which RC trained using the latest data up to a day predicts confirmed cases on the next day^35^. Here we demonstrate daily forecast predictions by using our PRC simulator. We used real-world data on daily confirmed COVID-19 cases from January 1, 2022, to June 29, 2022, spanning 180 days, in major Japanese cities such as Hokkaido, Tokyo, Osaka, and Fukuoka. This data was sourced from the Japanese Ministry of Health^36^. The preprocessed data $A'' = \lbrace a''_0, a''_1, ..., a''_{179}\rbrace$ with a total of 180 time steps were prepared by taking the weekly average and normalizing from 0.0 to 1.0 (see the Methods section for more details).

For the daily ahead forecast, inference and learning were implemented at each time step, and the readout was trained at each step using the latest data. During the inference at time step $\tau$, the prediction $y_\tau$ of the data $a_\tau$ is generated by the PRC system from the input data $x_\tau = a_{\tau -1}$. During learning at time step $\tau$, the readout was trained through batch learning to minimize the error between the ground truth $\lbrace a''_1, a''_2, ..., a''_\tau \rbrace$ and prediction $\lbrace y_1, y_2, ..., y_\tau \rbrace$. After repeating these processes for all time steps, RMSE and NRMSE between the forecast and preprocessed real values were obtained to evaluate forecasting capability.

**Figure 4:**
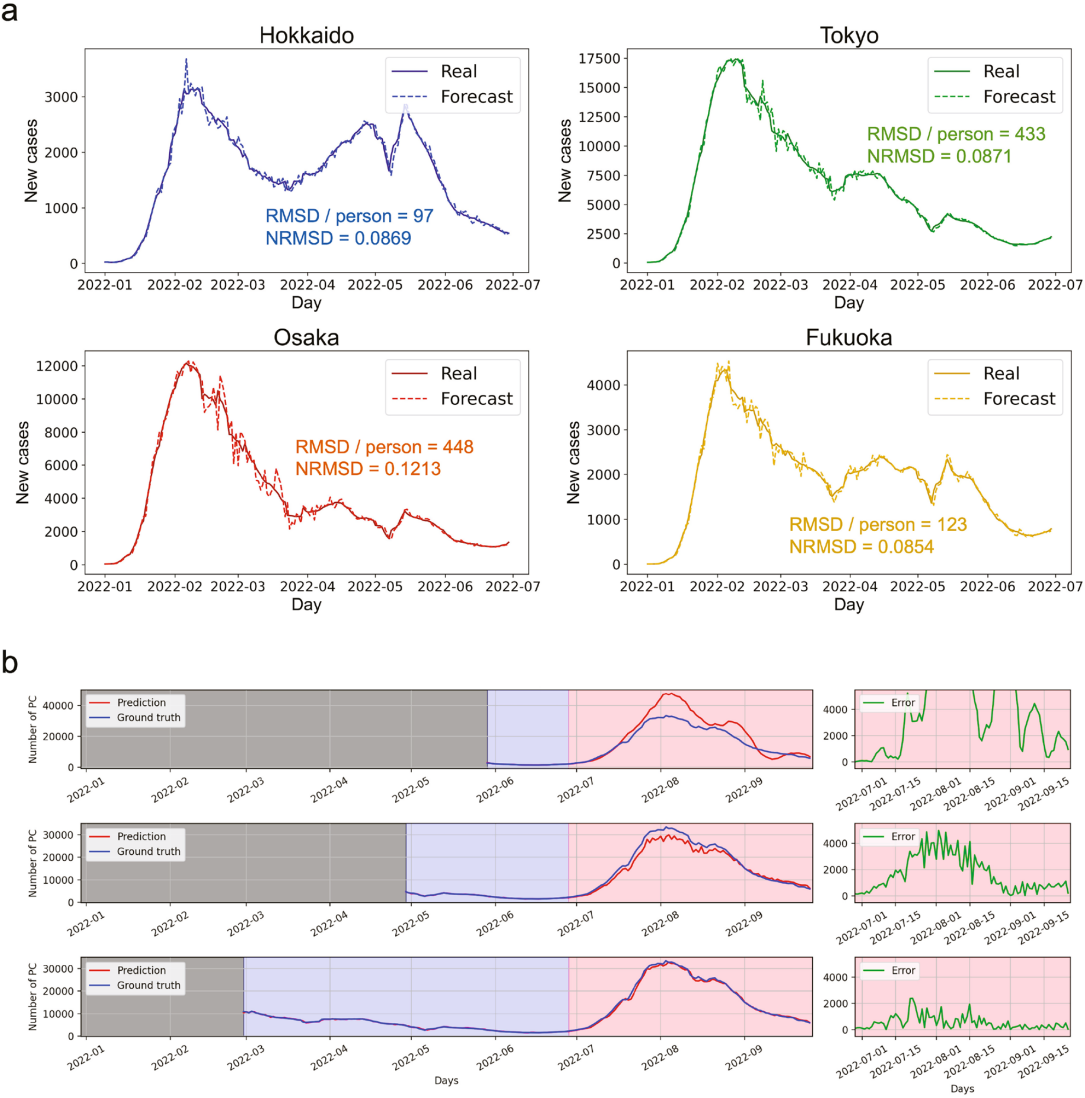
Daily ahead forecast of confirmed COVID-19 cases by the developed PRC simulator. (**a**) Comparison between the four prefectures in Japan; Hokkaido, Tokyo, Osaka and Fukuoka, in which the readout was sequentially trained using the latest data. Real lines represent preprocessed actual values, while dotted lines depict their corresponding forecasts. (**b**) Transition of daily confirmed COVID-19 cases (blue line), its forecast (red line), and the error between them (green line). Top, middle, and bottom rows show the results in which the readout was trained for periods (blue area) of 30, 60, and 120 days, respectively.

Figure 4a shows the daily ahead forecast results, and the obtained demoralized RMSE and NRMSE for each prefecture are summarized in Table 2 (see Methods section for details on denormalization). For all prefectures, the overall trend of confirmed case transitions was accurately forecasted. Forecasts for Hokkaido and Fukuoka, which are less populated prefectures, showed remarkable accuracy with a small demoralized RMSE of approximately 100 people. The confirmed cases in highly-populated metropolises, such as Tokyo and Osaka, were also forecasted successfully, with a demoralized RMSE of approximately 450 people. However, the comparison of NRMSE between the prefectures shows that the forecast for Osaka was more challenging and unstable than that for the other prefectures, as shown in Fig. 4a, opening further discussion of data preprocessing and training methods.

**Table 2:** Errors of daily ahead COVID-19 forecast for four prefectures.

Prefecture	RMSE/person	NRMSE
Hokkaido	97	0.0869
Tokyo	433	0.0871
Osaka	448	0.1213
Fukuoka	123	0.0854

Training the readout at every time step can sometimes be inefficient in terms of energy consumption and real-time predictions. Therefore, the daily ahead forecast of the confirmed COVID-19 cases was attempted by applying a high generalization capability of the proposed PRC system; the simulator of the proposed PRC system was trained using only past real-world data over a certain period to learn the underlying trend behind the confirmed case transition, and evaluate its forecasting capability of the future data. Data spanning 985 days from January 16, 2020, to September 26, 2022, were utilized, with the period from June 29, 2022, to September 26, 2022, comprising 90 days, specifically designated for the evaluation. Figure 4b presents the prediction results when the readout was trained on 30, 60, and 120 days, with NRMSEs of 0.3725, 0.1258, and 0.0484, respectively. Employing extensive training data, the proposed PRC system demonstrated high accuracy in forecasting rapid increases in confirmed cases. The developed PRC system’s ability to accurately predict rapid increases in confirmed cases, using a substantial amount of training data, indicates its proficiency in learning underlying trends solely from past data.

## Methods

### Development of hardware prototype

An overview of the hardware prototype is illustrated in Fig. S1 in Supplementary Information, where the analogue SCR circuits were implemented on eight PCBs. The design of the analogue SCR circuit was based on the NGSPICE simulation using ideal elements. For the hardware prototype, an 8-bit DAC (PCF8591, NXP Semiconductors) was used to generate a random input sequence. The states of 50 reservoir nodes on a PCB were read sequentially in a time step by an ADC in a logic analyzer (Saleae Logic Pro 16, Saleae Inc.) with 12-bit resolution, while the analogue switches (CD4066B, Texas Instruments Inc.) were controlled by the microcontroller (Arduino Uno R3, Arduino S.R.L.). The reservoir node states were read from eight PCBs simultaneously, that is, 400 node states were read at every time step. $R_{cas}, R_{nl}$ and $C_{hold}$ were set to 1.6 k$\Omega$, 1 k$\Omega$, and 0.22 $\mu$F, respectively. $R_{in}$ values for each node circuit were randomly generated in the range of 3.6 ~ 36 k$\Omega$. For the p-and n-channel MOSFETs in a nonlinear circuit, ZVP2106A (Diodes Incorporated) and LND150 (Microchip Technology) were selected. The operational amplifier, LMC6482 (Texas Instruments, Inc.) was used.

### Training with linear regression

The RC output *Z* is calculated using the reservoir state matrix *V* and the readout vector *W*, including biases, as detailed in Eq. (2). By employing batch learning with linear regression, *W* is optimized to minimize the error between *Z* and the supervisor output *Y*, as demonstrated in Eq. (3).

2 $$\begin{aligned} Z= & {} VW =\left( \begin{array}{ccccc} 1 &{} v_{1,0} &{} v_{2,0} &{} \cdots &{} v_{400,0}\\ 1 &{} v_{1,1} &{} v_{2,1} &{} \cdots &{} v_{400,1}\\ \vdots &{} \vdots &{} \vdots &{} \ddots &{} \vdots \\ 1 &{} v_{1,T} &{} v_{2,T} &{} \cdots &{} v_{400,T}\\ \end{array} \right) \begin{pmatrix} w_{0} \\ w_{1} \\ \vdots \\ w_{400} \end{pmatrix} \end{aligned}$$

3 $$\begin{aligned} W= & {} V^{-1}Y \end{aligned}$$

### Prediction of NARMA10

NARMA10, which is time-series data showing chaotic behaviour, was generated using the following equation:

4 $$\begin{aligned} y_\tau = 0.3y_{\tau -1}+0.05y_{\tau -1}{\sum _{k=1}^{10}y_{\tau -k}}+1.5x_{\tau -1}x_{\tau -10}+0.1 \end{aligned}$$

Here, $y_\tau$ denotes the supervisor at time step $\tau$ generated from the input to the reservoir $x_\tau$. For this study, a uniform random input sequence ranging from 0 to 0.5 was generated. The evaluation of prediction error employs the RMSE and NRMSE, defined as follows, with the total number of time steps *T* considered during the evaluation phase:

5 $$\begin{aligned}{} & {} { \text {RMSE}}(\textit{Y},\textit{Z})=\sqrt{\frac{(\textit{Y}-\textit{Z})^2}{T}} \end{aligned}$$

6 $$\begin{aligned}{} & {} { \text {NRMSE}}(\textit{Y},\textit{Z})=\frac{{ \text {RMSE}}}{\bar{Y}} \end{aligned}$$

### Linear and nonlinear memory capacity (MC)

The supervisor $$Y_k$$ (delay steps $$k = 0, 1, 2,..., 100)$$ of the RC output *Z* was generated from the input *U*, and the linear MC $$(MC^1)$$, defined as the determination coefficient between $$Y_k$$ and *Z* after training the readout, was calculated as follows:

7 $$\begin{aligned} y_k= & {} u(t-k) \end{aligned}$$

8 $$\begin{aligned} MC^1= & {} \sum _{k=0}^{100}MC_k^1 = \sum _{k=0}\frac{cov^2(Y_k,Z)}{\sigma ^2(Y_k), \sigma ^2(Z)} \end{aligned}$$

In addition, a nonlinear MC was obtained using the equations mentioned in^37^.

9 $$\begin{aligned} P_n(x)= & {} \frac{1}{2^nn!}\frac{d^n}{dx^n}(x^2-1)^n, n = 0, 1, 2,... \end{aligned}$$

10 $$\begin{aligned} y_{\lbrace d_k \rbrace ^q}= & {} \prod _{k=0}^{100}P_{d_k}(u(t-k)), q = \sum _{k=0}^{100}d_k \end{aligned}$$

11 $$\begin{aligned} MC^q= & {} \sum _{\lbrace d_k \rbrace ^q}\frac{cov^2(Y_{\lbrace d_k \rbrace ^q},Z)}{\sigma ^2(Y_{\lbrace d_k \rbrace ^q}), \sigma ^2(Z)} \end{aligned}$$

$P_n(x)$ in Eq. (9) is a Legendre polynomial of nonlinear degree *n*. $\lbrace d_k \rbrace ^q$ indicates a set of elements ${d_0, d_1, d_2,..., d_{100}}$, where $d_k$ is an integer greater than or equal to zero. *q* denotes the degree of nonlinearity, and Eqs. (10) and (11) correspond to Eqs. (7) and (8), when $q = 1$. $\lbrace d_k \rbrace ^q$ indicates a set in which the sum of all the elements is equal to *q*. MC of nonlinear degree *q* is defined by Eq. (11), where the sum of the determination coefficients for all sets is obtained.

### COVID-19 data preparation and preprocessing

For real-world data, daily confirmed COVID-19 case counts from January 17, 2022, to May 16, 2022, in the prefectures of Hokkaido, Tokyo, Osaka, and Fukuoka, Japan, were obtained from the Japanese Ministry of Health^36^. The full progression of confirmed cases in each prefecture during this period is detailed in the Supplementary Information, were noticeable increases in confirmed cases are evident in every prefecture.

The obtained data *A* were used as discrete time-series data, and all the data $a_\tau$ at time step $\tau$ were pre-processed as follows:

12 $$\begin{aligned} a_\tau '= & {} \sum _{i=\tau -7}^\tau \frac{a_i}{7} \end{aligned}$$

13 $$\begin{aligned} a_\tau ''= & {} \frac{a_\tau '-a_{min}'}{a_{max}'-a_{min}'} \end{aligned}$$

Eq. (12) represents the weekly average smoothing introduced to remove noise from the data. After smoothing, the data were normalized, as shown in Eq. (13) to input it into the PRC system, where $a_{min}'$ and $a_{max}'$ denote the minimum and maximum values of smoothed data $A'$, respectively. Conversely, obtained output *Z* from the PRC system was denormalized to $Z'$ to obtain the RMSE with the actual data $A'$ as follows:

14 $$\begin{aligned} z_\tau ' = z_\tau (a_{max}'-a_{min}')+a_{min}' \end{aligned}$$

### Supplementary Information

Supplementary Information.

## Data Availability

All data and methods supporting this study’s conclusions are detailed in the main text and Supplementary Information. For additional data, please contact the corresponding authors.
