# Supplementary material for: Highly-integrable analogue reservoir circuits based on a simple cycle architecture
Source: Sci Rep. 2024 May 14;14:10966. doi: 10.1038/s41598-024-61880-z (PMC11094067; doi:10.1038/s41598-024-61880-z)
Supplement: Supplementary file 1 — Supplementary Information. [file 41598_2024_61880_MOESM1_ESM.pdf]

Supplementary Material

Highly-Integrable Analog Reservoir Circuits Based on a Simple Cycle Architecture

Yuki Abe, Kazuki Nakada, Naruki Hagiwara, Eiji Suzuki, Keita Suda,
Shin-ichiro Mochiduki, Yukio Terasaki, Tomoyuki Sasaki, Tetsuya Asai

1 Supplementary to the constructed prototype of PRC system

Over view of the constructed prototype PRC system is shown in Fig. S1

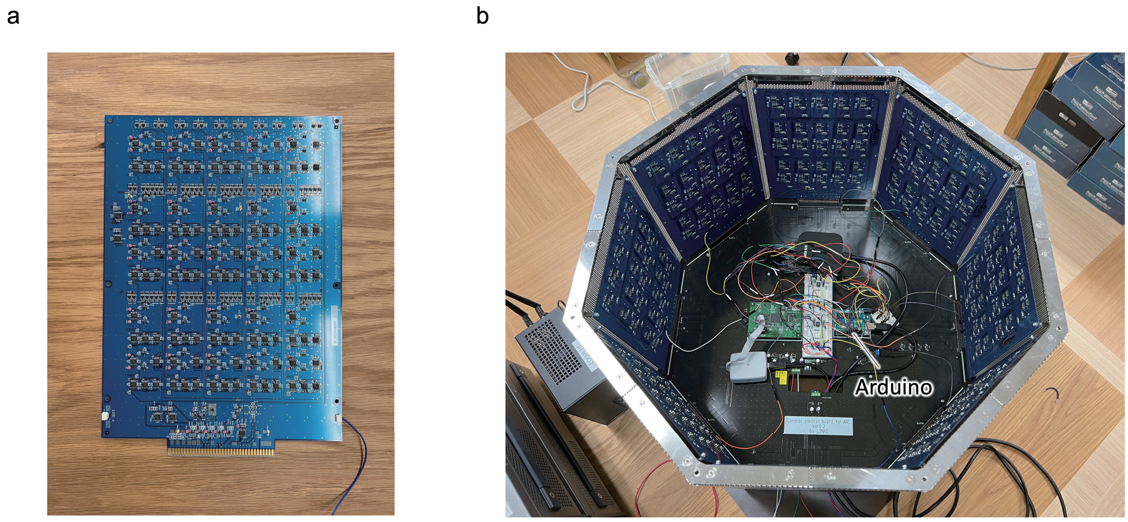

Figure S1: Over view of the constructed prototype PRC system. (a) Printed circuit board (PCB) (b) Whole system consisting of eight PCBs and a microcontroller (Arduino).

2 Supplementary to feasible study

I R&C circuit

A PRC system with simple R&C circuits as physical reservoir nodes was examined as a feasible study. The R&C circuit shown as Fig. S2a behaves as a node having a fading memory, in which the output potential changes time–dependently as Eq. 1 in the manuscript (Fig. S2b). Here, the PRC system was constructed on the circuit simulator, in which the 300 R&C circuits were connected in parallel as shown in Fig. S2c, and evaluated its performance through the benchmark tasks. All capacitance were set to $C = 10\mu$ F, and each resistance R was generated from uniformed random numbers in a range from 10k to 100 kΩ. These parameters were selected so that the time constant of each R&C circuit fell between 0.1 and 1.0 s. The results of benchmark tasks are shown in Fig. S2d–f, in which NRMSE of NARMA10 and linear MC were 0.1606 and 9.38, respectively, indicating a low performance as a reservoir. Notably, nonlinear MCs shown in Fig. S2f indicate that there is no nonlinear component in the system because the R&C circuit works linearly as Eq. (1) in the manuscript.

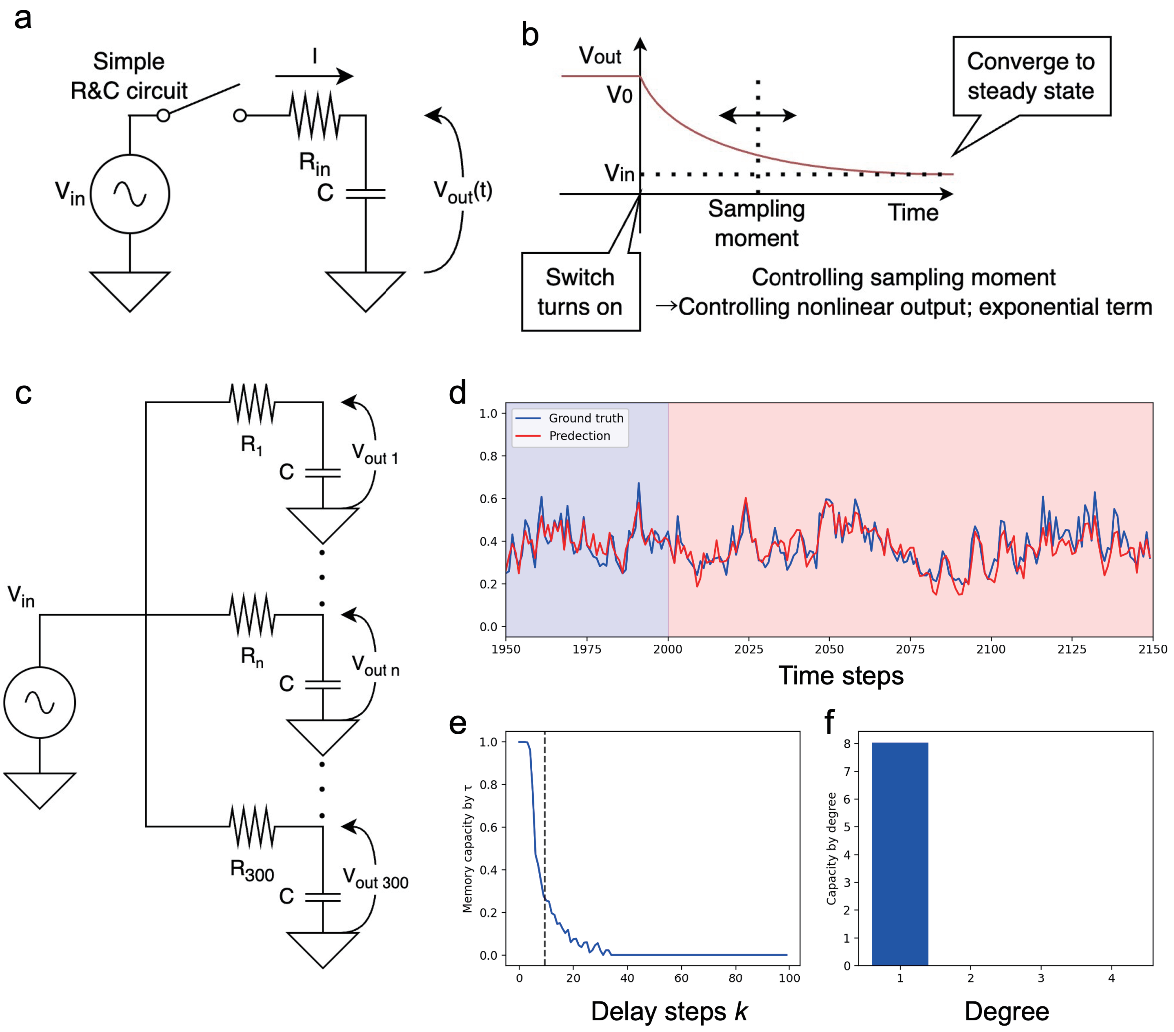

Figure S2: (a) Circuit diagram of a R&C circuit. (b) Schematic diagram of a transition of output potential V_{out}. (c) Circuit diagram of a PRC system, in which R&C circuits are connected in parallel. (d–f) Benchmark results of (d) NARMA10 prediction, (e) linear MC, and (f) nonlinear MC.

II Nonlinear circuit

To provide nonlinearities with the PRC system with the R&C circuit, introducing additional nonlinear circuits was examined. Here, a nonlinear circuit inspired by a horizontal resistor (HRes) circuit [1], which has a nonlinear I–V characteristic like a sigmoid function (Fig. S3a, c), was newly designed. A circuit diagram of the nonlinear circuit, which has a quite simple architecture containing only two MOSFETs, is shown in Fig. S3b. The changes from the HRes circuit are as follows.

1. Output of circuit is a voltage, not a current.

2. The number of voltage source was reduced to simplify the circuit configuration.
3. Input–output characteristic shows like a half-sigmoid function (Fig. S3d).

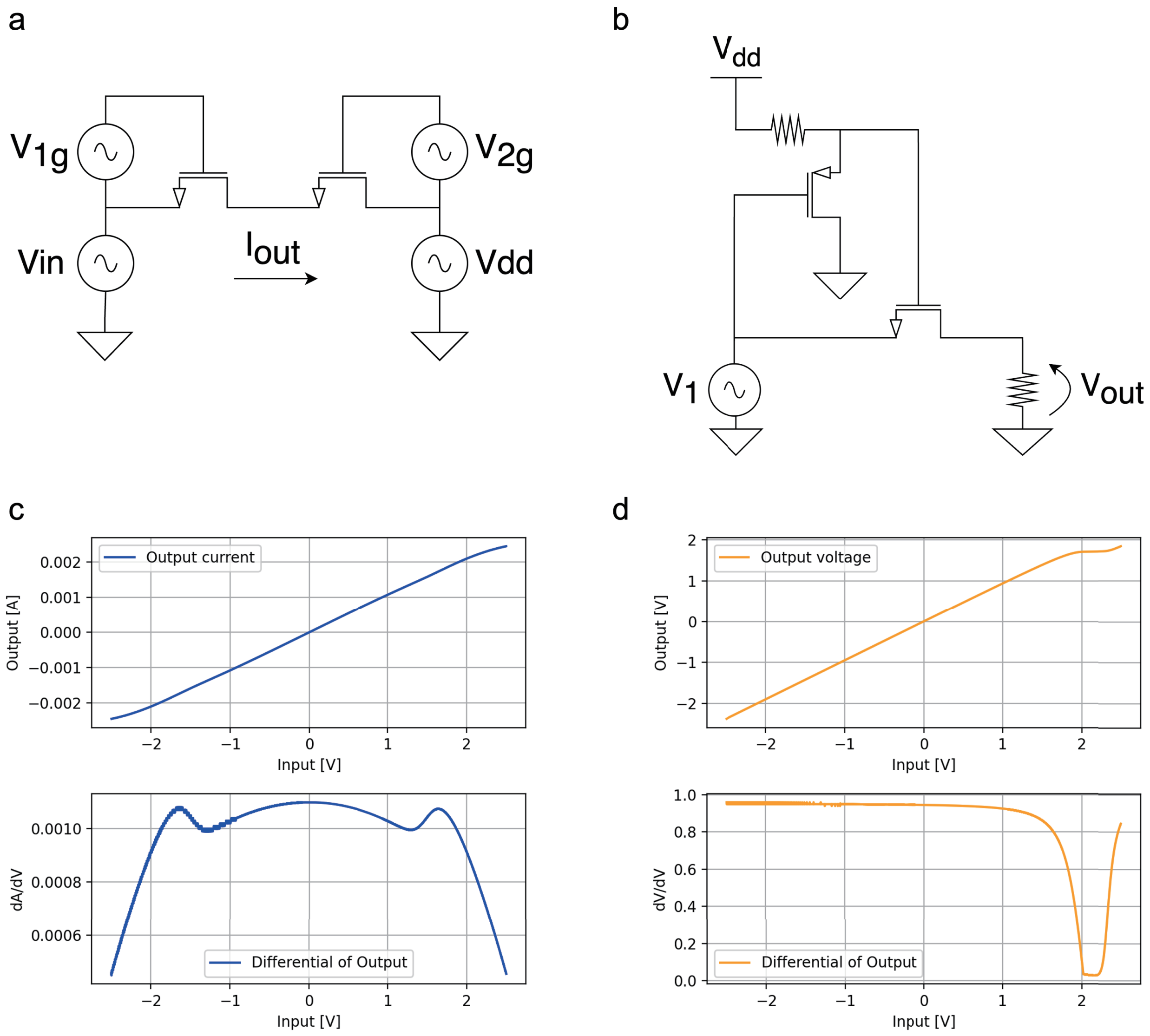

Figure S3: Circuit diagrams of (a) a horizontal resistor circuit and (b) our proposed nonlinear circuit. Input–output characteristics of (c) the horizontal resistor circuit ($V_{1g} = V_{2g} = 0.1$) and (d) our proposed nonlinear circuit.

3 Supplementary to performance evaluation

I Comparison with other PRC systems

Benchmarks such as linear MC and NARMA10 task used in this study have often been used and reported to measure the performance of PRC systems on experiments or simulations. Comparisons of linear MC and errors obtained at NARMA10 task between our proposed and other PRC systems are summarized in Table S1. Note that the way to calculating linear MC differs between the papers depending on the type of input signal (a uniform random number or a binary random number). Linear MCs obtained by using a binary random input sequence, not an uniform random input used in this study, are indicated by * in Table S1. These comparisons will be helpful to compare their performances in broad strokes.

II Dependence of ADC resolution

In this study, 12–bit ADCs were used for reading the reservoir states. However, it is known that the quantization errors of ADC can cause the performance degradation of PRC [9]. Table S2 shows the linear and nonlinear MC

Table S1: Example of a lengthy table which is set to full textwidth

Reference	Medium of physical reservoir	Linear MC	Prediction error of NARMA10 task	Simulation / Experiment
[2, 3]	Optical device	31.9	N/A	Experiment
[4]	Analog circuit	N/A	0.18 (NRMSE)	Simulation
[5]	Molecular network	33.5 *	N/A	Experiment
[6]	Analog circuit	120<= *	0.055 (NRMSE)	Simulation
[7]	Analog circuit	N/A	0.0683 (NRMSE)	Simulation
[8]	Spin device	42 *	N/A	Experiment
[3]	Optical device	21.14	0.107 (NMSE)	Experiment
This study	Analog circuit	58.44	0.0973 (NRMSE), 0.0397 (RMSE)	Simulation
		45.73	0.0861 (NRMSE), 0.0351 (RMSE)	Experiment

obtained through simulations with different ADC resolutions, showing both linear and nonlinear MC deteriorated as the number of ADC bits decreased. Notably, the nonlinear MC deteriorated significantly. To maximize the performance of this PRC system, it is necessary to use an ADC with a sufficiently high resolution.

Table S2: Dependence of ADC resolution on linear and nonlinear memory capacity

Quantization level	Linear	Quadratic	Cubic
8 bit	39.80	0.00	0.00
12 bit	57.92	24.65	0.00
16 bit	71.51	256.68	0.00

4 Supplementary to prepared data of the daily confirmed COVID–19 cases

Figure S4 shows the whole data of the daily confirmed COVID–19 cases at four prefectures in Japan, which is used for demonstrations in this study.

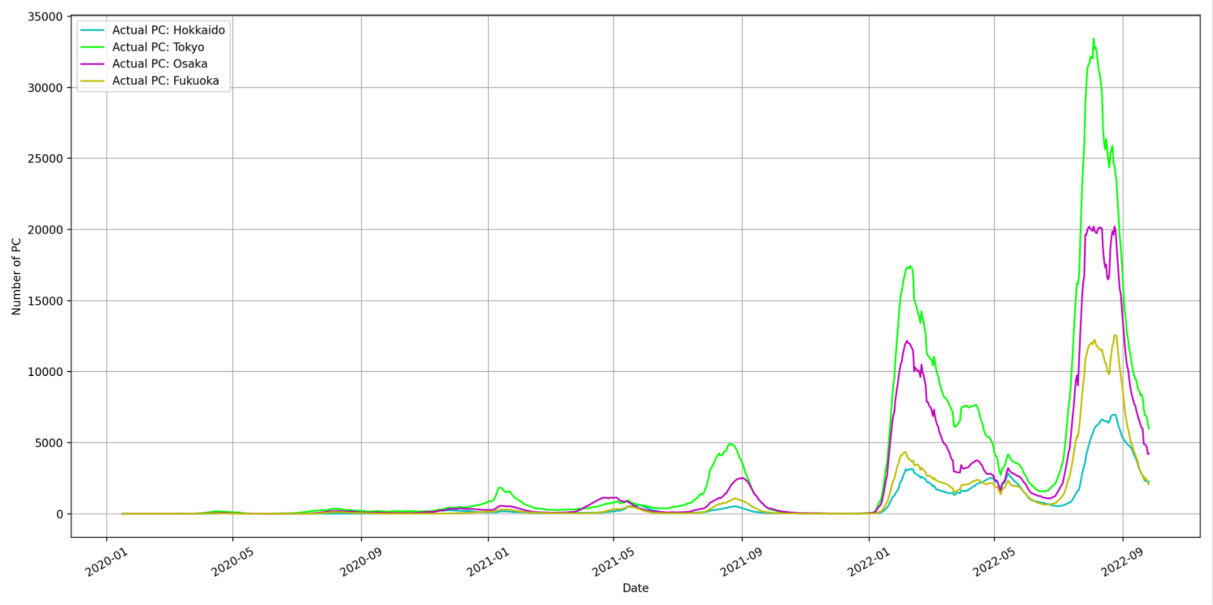

Figure S4: Transitions of the daily confirmed COVID–19 cases during a period from 17/1/2022 to 16/5/2022 at Hokkaido, Tokyo, Osaka, and Fukuoka.

References

[1] Mohammed Ismail Carver Mead. *Analog VLSI Implementation of Neural Systems.* Springer New York, 2012.

[2] Yvan Paquot, François Duport, Anteo Smerieri, J. Dambre, Benjamin Schrauwen, M. Haelterman, and Serge Massar. Optoelectronic reservoir computing. *Scientific Reports*, Vol. 2, , 11 2011.

[3] Quentin Vinckier, François Duport, Anteo Smerieri, Kristof Vandoorne, Peter Bienstman, Marc Haelterman, and Serge Massar. High-performance photonic reservoir computer based on a coherently driven passive cavity. *Optica*, Vol. 2, No. 5, pp. 438–446, May 2015.

[4] Lennert Appeltant, Miguel Soriano, Guy Van der Sande, Jan Danckaert, Serge Massar, J. Dambre, Benjamin Schrauwen, Claudio Mirasso, and Ingo Fischer. Information processing using a single dynamical node as complex system. *Nature communications*, Vol. 2, p. 468, 09 2011.

[5] Yuki Usami, Bram van de Ven, Dilu G. Mathew, Tao Chen, Takumi Kotooka, Yuya Kawashima, Yuichiro Tanaka, Yoichi Otsuka, Hiroshi Ohoyama, Hakaru Tamukoh, Hirofumi Tanaka, Wilfred G. van der Wiel, and Takuya Matsumoto. In-materio reservoir computing in a sulfonated polyaniline network. *Advanced Materials*, Vol. 33, No. 48, p. 2102688, 2021.

[6] Xiangpeng Liang, Yanan Zhong, Jianshi Tang, Zhengwu Liu, Peng Yao, Keyang Sun, Qingtian Zhang, Bin Gao, Hadi Heidari, He Qian, and Huaqiang Wu. Rotating neurons for all-analog implementation of cyclic reservoir computing. *Nature Communications*, Vol. 13, , 03 2022.

[7] Kangjun Bai and Yang Yi. Dfr: An energy-efficient analog delay feedback reservoir computing system for brain-inspired computing. *J. Emerg. Technol. Comput. Syst.*, Vol. 14, No. 4, dec 2018.

[8] Ryosho Nakane, Akira Hirose, and Gouhei Tanaka. Spin waves propagating through a stripe magnetic domain structure and their applications to reservoir computing. *Phys. Rev. Res.*, Vol. 3, p. 033243, Sep 2021.

[9] Miguel C. Soriano, Silvia Ortín, Lars Keuninckx, Lennert Appeltant, Jan Danckaert, Luis Pesquera, and Guy van der Sande. Delay-based reservoir computing: Noise effects in a combined analog and digital implementation. *IEEE Transactions on Neural Networks and Learning Systems*, Vol. 26, No. 2, pp. 388–393, 2015.
